# Effective force control by muscle synergies

**DOI:** 10.3389/fncom.2014.00046

**Published:** 2014-04-17

**Authors:** Denise J. Berger, Andrea d'Avella

**Affiliations:** Laboratory of Neuromotor Physiology, Santa Lucia FoundationRome, Italy

**Keywords:** non-negative matrix factorization, isometric force, reaching movements, myoelectric control, modularity, electromyography

## Abstract

Muscle synergies have been proposed as a way for the central nervous system (CNS) to simplify the generation of motor commands and they have been shown to explain a large fraction of the variation in the muscle patterns across a variety of conditions. However, whether human subjects are able to control forces and movements effectively with a small set of synergies has not been tested directly. Here we show that muscle synergies can be used to generate target forces in multiple directions with the same accuracy achieved using individual muscles. We recorded electromyographic (EMG) activity from 13 arm muscles and isometric hand forces during a force reaching task in a virtual environment. From these data we estimated the force associated to each muscle by linear regression and we identified muscle synergies by non-negative matrix factorization. We compared trajectories of a virtual mass displaced by the force estimated using the entire set of recorded EMGs to trajectories obtained using 4–5 muscle synergies. While trajectories were similar, when feedback was provided according to force estimated from recorded EMGs (EMG-control) on average trajectories generated with the synergies were less accurate. However, when feedback was provided according to recorded force (force-control) we did not find significant differences in initial angle error and endpoint error. We then tested whether synergies could be used as effectively as individual muscles to control cursor movement in the force reaching task by providing feedback according to force estimated from the projection of the recorded EMGs into synergy space (synergy-control). Human subjects were able to perform the task immediately after switching from force-control to EMG-control and synergy-control and we found no differences between initial movement direction errors and endpoint errors in all control modes. These results indicate that muscle synergies provide an effective strategy for motor coordination.

## Introduction

How the CNS coordinates a large number of muscles to control forces and movements is a long standing issue in neuroscience. Muscle synergies, coordinated recruitment of groups of muscles with specific activation balances or temporal profiles, have been proposed as building blocks employed by the CNS to simplify the generation of forces or movements (Jacobs and Macpherson, 1996; Tresch et al., 1999; Bizzi et al., 2002; d'Avella et al., 2003; Flash and Hochner, 2005; Giszter et al., 2007; Ting and McKay, 2007; Bizzi et al., 2008; d'Avella and Pai, 2010; Lacquaniti et al., 2012; Bizzi and Cheung, 2013; d'Avella and Lacquaniti, 2013). A small number of muscle synergies, identified by multidimensional factorization techniques such as non-negative matrix factorization (NMF) (Lee and Seung, 1999), independent component analysis (ICA) (Bell and Sejnowski, 1995), and other iterative algorithms (d'Avella and Tresch, 2002; Tresch et al., 2006; Omlor and Giese, 2011), have been shown to explain a large fraction of the variation in the muscle patterns in a variety of vertebrate species (Tresch et al., 1999; Saltiel et al., 2001; d'Avella et al., 2003; Hart and Giszter, 2004; Ivanenko et al., 2004; Cheung et al., 2005; Ting and Macpherson, 2005), across different behaviors and experimental conditions (d'Avella and Bizzi, 2005; Cappellini et al., 2006; d'Avella et al., 2006, 2008, 2011; Ivanenko et al., 2007; Torres-Oviedo and Ting, 2007; Overduin et al., 2008; Torres-Oviedo and Ting, 2010; Dominici et al., 2011; Hug et al., 2011; Chvatal and Ting, 2012; Frere and Hug, 2012; Roh et al., 2012; Chvatal and Ting, 2013; d'Andola et al., 2013; Gentner et al., 2013). These observations provide support to the existence of muscle synergies as neural control strategy employed by the CNS for motor coordination. However, they do not directly demonstrate that a small number of synergies is sufficient to generate the functional output of muscle patterns, i.e., the forces or movements necessary for accomplishing a task (Alessandro et al., 2013). Thus, in order to validate muscle synergies as a neural control strategy their functional consequences need to be investigated.

Torres-Oviedo et al. (2006) first investigated the functional consequences of muscle synergies by extracting synergies simultaneously from EMGs and foot contact forces during postural responses to multidirectional stance perturbations in cats. Such functional muscle synergies were able to explain both muscle activation patterns and endpoint forces in a range of postural configurations, thereby supporting a functional role for the synergies. A common set of functional muscle synergies were then also found to explain different types of postural responses in human subjects (Chvatal et al., 2011). Furthermore, forward dynamics simulations using a musculoskeletal model of the human trunk, pelvis, and legs have shown that a small number of muscle synergies are sufficient to perform the basic sub-tasks of walking in two (Neptune et al., 2009) and three (Allen and Neptune, 2012) dimensions. More recently de Rugy et al. (2013) addressed the question of the functional consequences of muscle synergies in the context of isometric force generation at the wrist. They estimated forces as a linear function of EMGs recorded from five wrist muscles. Subject used such estimated forces to perform a force reaching task. de Rugy and collaborators then compared the forces estimated using EMGs with the forces estimated using synergies extracted from the EMGs. Four synergies explained most of the variation in the muscle patterns but they were not able to accurately reproduce the forces estimated using the recorded EMGs. However, estimated and real forces were not compared and it is not clear whether the apparent inaccuracy of the forces estimated using the synergies is specific to the wrist system.

Here, we extended the analysis of de Rugy et al. (2013) to a more complex and redundant system. We recorded muscle activity from 13 arm and shoulder muscles in humans performing a force reaching task in which a cursor in a virtual environment was displaced according to either the recorded isometric force (force-control) or the force estimated from the recorded EMGs (EMG-control). We compared the cursor trajectories executed during EMG-control with the trajectories reconstructed using synergies, as in de Rugy et al. (2013). However, we also performed two additional comparisons. First, we investigated the reconstruction of trajectories executed in force-control using synergies and individual muscles. Second, to explicitly validate the synergy hypothesis as a possible control principle, we directly tested whether subjects were able to control movements with synergies. We let subjects perform the force reaching task in force-control, in EMG-control, and in synergy-control, i.e., by projecting online each sample of the recorded muscle patterns in the synergy space, and we compared their performances across the three conditions.

We found that, across subjects, 4–5 synergies could capture adequately the EMG data variation but they were not sufficient to reconstruct the trajectories executed in EMG-control with the same endpoint accuracy, thus extending to the arm the results obtained for the wrist by de Rugy et al. (2013). However, when we compared the reconstructions of trajectories executed in force-control using individual muscles and synergies we did not find any significant difference in several performance measures. These results demonstrate that individual muscles and synergies perform equally well in the prediction of the applied forces that were generated by human subjects. Finally, we found that humans were not only able to perform the task immediately after switching from force-control to EMG-control and synergy-control, but they also did not show any differences in performance between the three conditions. These results demonstrate that human subjects can achieve similar performances in an isometric reaching task using a small number of synergies and using individual muscles.

## Materials and methods

We asked naïve participants to reach targets on a virtual desktop by displacing a cursor (i.e., a virtual spherical handle) according to: (1) the force applied on a physical handle (force-control); (2) the force estimated from the EMG activity recorded from many shoulder and arm muscles (myoelectric or EMG-control); (3) the force estimated from the combination of the recorded EMG signals through a set of muscle synergies (synergy-control). Initially the reaching task was performed under force-control and, for each individual participant, the force and EMG data collected were used to estimate an EMG-to-force matrix by multiple linear regressions. EMG data collected during force-control or during EMG-control were also used to identify muscle synergies by non-negative matrix factorization. Such synergies were then used to reconstruct cursor trajectories executed in force- and EMG-control and to execute trajectories in synergy-control.

### Participants

14 right-handed naïve subjects (mean age 26.0 years, *SD* 3.5, age range 20–34, 9 females) participated in the experiments after giving written informed consent. All procedures were conducted in conformance with the Declaration of Helsinki and were approved by the Ethical Review Board of Santa Lucia Foundation.

### Experimental set-up

**Figure 1:**
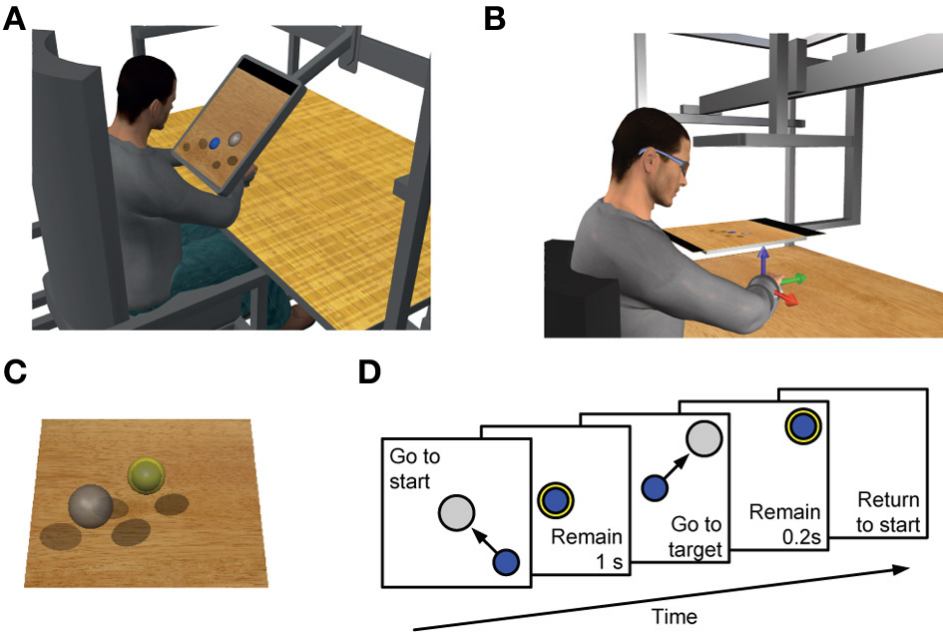
**Experimental setup and trial sequence**. Subjects sat in front of a desktop and applied forces on a transducer attached to a forearm, wrist, and hand splint. In Experiment 1 a LCD monitor **(A)** and in Experiment 2 a mirror **(B)** occluded the subject's hand and displayed a virtual scene co-located with the real desktop. **(C)** Transparent spheres positioned on a horizontal plane with centers at the same height as the center of the palm indicated force targets that the subjects were instructed to reach with a smaller spherical cursor moving on the same plane according to the force applied (force-control) or estimated from EMGs recorded from 13 arm and shoulder muscles (EMG-control, see Materials and Methods), or estimated from recorded EMGs recombined using a set of synergies extracted using NMF (synergy-control, see Materials and Methods). **(D)** Subjects were instructed to perform a center-out reaching task in which they had to maintain the cursor in a central start location for 1 s, reach a target as soon as it appeared at one of 8 peripheral locations, and maintain the cursor at the target for 0.2 s. **(A)** and **(C)** adapted from Berger et al. (2013); **(B)** adapted from Borzelli et al. (2013).

Subjects sat in front of a desktop on a racing car seat with their torso immobilized by safety belts, their right forearm inserted in a splint, immobilizing hand, wrist, and forearm. The center of the palm was aligned with the body midline at the height of the sternum and the elbow was flexed by approximately 90°. The subjects' view of their hand was either occluded by a 21-inch LCD monitor inclined with its surface approximately perpendicular to the subjects' line of sight when looking at their hand (Figure 1A) during Experiment 1 (see Experimental Protocols below) or by a mirror displaying the virtual scene co-located with the real desktop positioned above the mirror (Figure 1B) during Experiment 2. After calibration, the monitor could display a virtual desktop matching the real desktop, a spherical cursor matching, at rest, the position of the center of the palm and moving on a horizontal plane, and spherical targets on the same plane (Figure 1C). A steel bar at the base of the splint was attached to a 6-axis force transducer (Delta F/T Sensor, ATI Industrial Automation, Apex, NC, USA) positioned below the desktop to record isometric forces and torques. Surface electromyographic (EMG) activity was recorded from the following 13 muscles acting on the shoulder and elbow: brachioradialis (BracRad), biceps brachii short head (BicShort), biceps brachii long head (BicLong), triceps brachii lateral head (TriLat), triceps brachii long head (TriLong), infraspinatus (InfraSp), anterior deltoid (DeltA), middle deltoid (DeltM), posterior deltoid (DeltP), pectoralis major (PectMaj), teres major (TerMaj), latissimus dorsi (LatDorsi), middle trapezius (TrapMid). EMG activity was recorded with active bipolar electrodes (DE 2.1, Delsys Inc., Boston, MA), band-pass filtered (20–450 Hz) and amplified (gain 1000, Bagnoli-16, Delsys Inc.). Force and EMG data were digitized at 1 KHz using an A/D PCI board (PCI-6229, National Instruments, Austin, TX, USA). The virtual scene was rendered by a PC workstation with a refresh rate of 60 Hz using custom software. In Experiment 2 the scene was rendered stereoscopically using a 3D graphic card (Quadro Fx 3800, NVIDIA Corporation, Santa Clara, CA, USA) and shutter glasses (3D Vision P854, NVIDIA). Cursor position information was processed by a second workstation running a real-time operating system and transmitted to the first workstation. Cursor motion was simulated in real time using an adaptive mass-spring-damper (MSD) filter (Park and Meek, 1995). Either the actual force recorded by the transducer (force-control), or the force estimated in real-time from the recorded and rectified EMGs (myoelectric or EMG-control) using a linear mapping (EMG-to-force matrix, *see below*), or the force estimated in real-time from synergies using the EMG-to-force matrix (synergy-control, *see below*), was applied to a virtual mass attached to a reference position through a critically damped spring. The position of the cursor corresponded to the position of the virtual mass. The reference position matched the position of the center of the palm. To maintain fast response to changes in force while reducing the effect of myoelectric noise, the simulated mass was adapted dynamically according to the time derivative of the applied force magnitude. Further details can be found in Berger et al. (2013).

### Experimental protocols

In all experiments subjects initially performed two blocks of trials in force-control. In the first force-control block, the mean maximum voluntary force (MVF) along 8 directions (separated by 45 deg) in the horizontal plane was estimated as the mean of the maximum force magnitude recorded across 16 trials in which subjects were instructed to generate maximum force in each direction. Subjects were then instructed to move the cursor quickly from the rest position to a target in one of the 8 directions by applying forces on the splint. At the beginning of each trial (Figure 1D) subjects were requested to maintain the cursor within a transparent sphere at the central start position for 1 s (tolerance of 2% MVF). Next, a *go* signal was given by displaying a transparent target sphere while the start sphere disappeared. Subjects were instructed to reach the target as quickly as possible and to remain there for 0.2 s (tolerance of 2% MVF). After successful target acquisition the cursor and the target disappeared indicating the end of the trial. Trials had to be completed within 2 s from the *go* signal. In the second force-control block subjects performed 72 trials to targets positioned at force magnitudes corresponding to 10, 20, and 30% of MVF (random order within cycles of 8 directions). After this block there was a 5 min pause to process the recorded data in order to construct the myoelectric controller. All subsequent blocks consisted of 24 trials with targets at 20% MVF in random order within cycles of 8 directions.

#### Experimental protocol 1

Eight participants (numbered from 1 to 8) performed this protocol. Data collected in force-control and EMG-control mode in our previous study (Berger et al., 2013) were used in this study to reconstruct cursor trajectories with synergies. After two initial blocks of trials in force-control the rest of the experiment was performed in EMG-control. For the purpose of the present analysis we only considered the force-control block and the second EMG-control block. Further details of the experimental protocol are described elsewhere (Berger et al., 2013).

#### Experimental protocol 2

Six participants (numbered from 9 to 14) performed this protocol. After the initial two blocks of trials performed in force-control, the system switched to synergy-control, using the subject specific synergies from the initial force-control block (synergy-control, *see below*). After 6 blocks of synergy-control, three blocks of force-control were introduced; this was followed by 6 blocks of EMG-control. At the end of the experiment a final block in force-control was performed.

### EMG-to-force mapping

**Figure 2:**
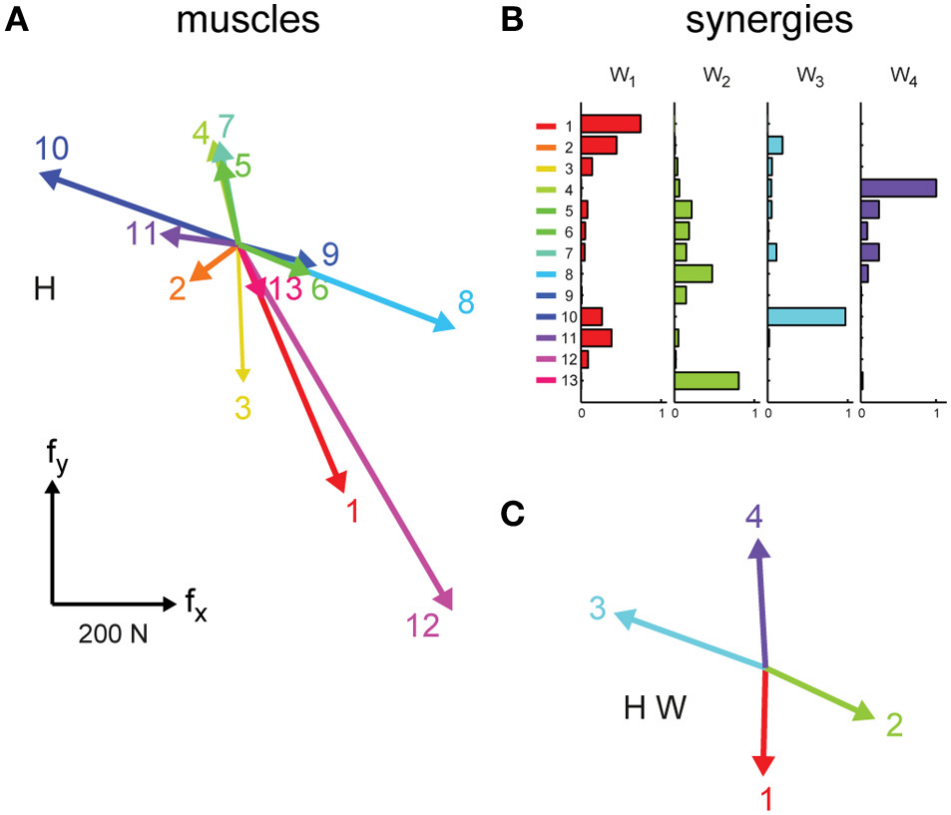
**EMG-to-force mapping and synergies**. **(A)** EMG-to-force matrix H estimated for subject 2 in Experiment 1 from EMG and force data recorded during the performance of the task in force-control. Each column of H, representing the planar force generated by one muscles, is illustrated by a colored arrow (1: brachioradialis, 2: biceps brachii short head, 3: biceps brachii long head, 4: triceps brachii lateral head, 5: triceps brachii long head, 6: infraspinatus, 7: anterior deltoid, 8: medial deltoid, 9: posterior deltoid, 10: pectoralis major, 11: teres major, 12: latissimus dorsi, 13: middle trapezius). **(B)** Muscle synergies (matrix W) identified by non-negative matrix factorization from the EMG data of subject 2 collected in the force-control block of Experiment 1. Each column of W, a vector specifying a specific pattern of relative level of muscle activation, is illustrated by color-coded horizontal bars. **(C)** Forces associated to the muscle synergies [W, shown in **(B)**] through the EMG-to-force matrix [H, shown in **(A)**], i.e., the columns of the matrix product H W (synergy-to-force matrix). Adapted from Berger et al. (2013).

If the arm is in a fixed posture, the force generated at the hand is approximately a linear function of the activation of muscles acting on shoulder and elbow:

(1) $$f=Hm+e_{f}$$

where **f** is the generated 2-dimensional force vector, **m** is the 13-dimensional vector of muscle activations, and **H** is a matrix relating muscle activation to force (dimensions 2 × 13), and **e_f_** is a 2-dimensional vector of force residuals. The EMG-to-force matrix (**H**) was estimated using multiple linear regressions of each applied force component, low-pass filtered (2nd order Butterworth, 1 Hz cutoff), with EMG signals recorded during the initial force-control block (dynamic phase, i.e., time from target go until the target has been reached), low-pass filtered (2nd order Butterworth, 5 Hz cutoff), and normalized to the maximum EMG activity during the generation of MVF. We verified that the choice of filter parameters for the estimation of the **H** matrix did not affect the quality of the force reconstruction during EMG-control by investigating different force and EMG cutoff frequencies. Figure 2A illustrates an example of the columns of the EMG matrix (i.e., the force associated to each muscle, **h_i_**) estimated in subject 2.

### Synergy extraction

Muscle synergies were identified by NMF (Lee and Seung, 1999) from EMG patterns from the go signal to target acquisition (dynamic phase):

(2) $$m=Wc+e_{m}$$

with **W** a *M* × *N* synergy matrix whose columns are vectors specifying relative muscle activation levels (*N* number of synergies, and *M* number of muscles), and **c** a *N*-dimensional synergy activation vector, and **e**_**m**_ is a *M*-dimensional vector of muscle activation residuals. For the comparison between trajectories executed in EMG-control and their reconstruction using synergies EMG patterns recorded during EMG-control were used for synergy extraction. For the comparison of trajectories from data collected during force-control EMG patterns recorded during force-control were used for synergy extraction. EMG patterns were first low-pass filtered (2nd order Butterworth filter, 5 Hz cutoff frequency) and rectified, their baseline noise level was then subtracted, and finally they were normalized to the maximum EMG activity of each muscle recorded during the generation of MVF. Baseline noise was estimated at the beginning of the experiment and updated periodically throughout the experiment while the subject was relaxed. For each possible *N* from 1 to *M*, the extraction algorithm was repeated 10 times and the repetition with the highest reconstruction R^2^ was retained. Figure 2B illustrates an example of the set of 4 synergies extracted in subject 2.

### Number of synergies

For each subject the number of synergies adequately capturing the EMG data (*N*) was selected according to the fraction of data variation explained, defined as R^2^_EMG_ = 1-SSE_EMG_/SST_EMG_, where SSE_EMG_ is the sum of the squared muscle activation residuals and SST_EMG_ is the sum of the squared residuals of the muscle activation from its mean vector. We considered two criteria. The first criterion was a threshold of 0.9 on R^2^_EMG_. The second criterion was based on the detection of a change in slope in the curve of the *R*^2^ value as a function of *N*. A series of linear regressions were performed on the portions of the curve included between *N* and its last point (*M*). *N* was then selected as the minimum value for which the mean squared error of the linear regression was less than 10^−4^. In case of mismatch between the two criteria, the larger *N* was chosen.

### EMG- and synergy-control

Output forces **f** during EMG-control were computed using the EMG-to-force mapping (**H**) and the recorded muscle activity **m** (compare EMG-to-force mapping), i.e., by

(3) f=H m

thus allowing for individual muscle control. During synergy-control muscle activity was substituted by the product of the initially extracted subject-specific synergies (**W**) and estimated synergy coefficients (**ĉ**), i.e., by **f = H W ĉ**, where **H W** is the synergy-to-force mapping (illustrated in Figure 2C for subject 2). Synergy coefficients were estimated by projecting recorded muscle activity onto the synergy space, i.e., by **ĉ = W^+^ m**, where **W^+^** is the pseudo inverse of **W**, corresponding to estimating **ĉ** from **m** as least squares solution of **m** = **W c**. Thus, during synergy-control output forces were computed as:

(4) $$f=H\;W\;W^{+}m$$

### Trajectory reconstruction using EMGs and synergies

Cursor trajectories executed in EMG-control, i.e., computed online as the displacement of a virtual mass-spring-damper system under the force estimated by Equation 3, were reconstructed using synergies, i.e., by computing offline how the cursor would have been displaced using the forces computed by Equation 4. Similarly, cursor trajectories executed in force-control, i.e., computed online as the displacement of a virtual mass-spring-damper system under the recorded force, were reconstructed using EMGs, i.e., using the forces computed by Equation 3, and synergies, i.e., using the forces computed by Equation 4.

### Performance measures

We compared cursor trajectories driven by EMGs, i.e., either executed in EMG-control (EC) or reconstructed by EMGs (ER) during force-control, with trajectories reconstructed by synergies (SR) during either EMG- or force-control, by assessing the fraction of EMG-driven trajectory variation explained, R^2^_traj_ = 1−SSE_traj_/SST_traj_, where SSE_traj_ is the sum of the squared trajectory residuals and SST_traj_ is the sum of the squared residuals of the EMG-driven trajectory from its mean vector. We also compared cursor trajectories computed from recorded forces with trajectories reconstructed by EMGs (ER) or synergies (SR) during force-control by assessing the fraction of force-driven trajectory variation explained with a similarly defined R^2^_traj−force_ measure. Both such measures quantified the similarity of the entire time course of two sets of trajectories. We then also compared performances at the beginning of the movement, quantifying an initial angle error, and at the end of each movement, quantifying an endpoint error. Initial angle error was defined as the angular deviation of the initial movement direction of the cursor with respect to target direction. The angular deviation was computed as *abs*(ϑ_target_−ϑ_cursor_), where ϑ_target_ is the target direction and ϑ_cursor_ is the direction of the displacement between the position of the cursor at movement onset and at the first following peak of its tangential velocity. Taking the absolute value avoided cancellations when averaging the values of the angular deviations across targets with different signs for the difference between target direction and cursor initial direction. Endpoint error was defined as the Euclidean distance, normalized to target distance from the origin, between the target position and the mean cursor position during the 0.2 s following the cursor's entrance into the target region. Finally, in Experiment 2, we compared the fraction of unsuccessful trials, i.e., the fraction of trials in which the cursor did not reached and remained in the target within the instructed time intervals, during the task execution in EMG-control and in synergy-control.

### Statistical analysis

Differences in performance measures were assessed either by *t-test* statistics (paired, two-tailed) if the data were distributed normally (according to a Lilliefors test) or by Wilcoxon ranksum test otherwise.

## Results

### Synergy reconstruction of cursor trajectories during EMG-control

**Figure 3:**
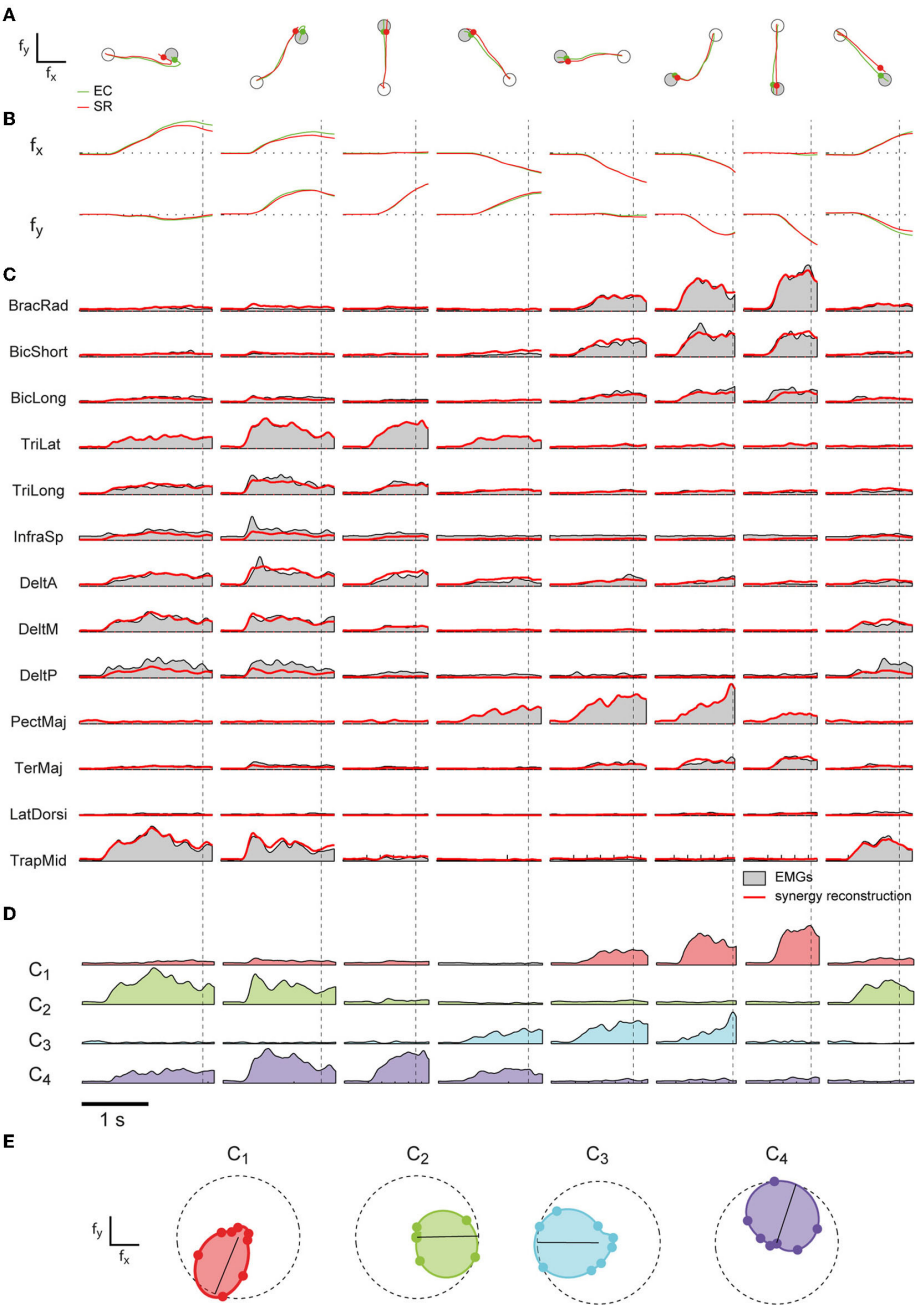
**Examples of cursor trajectories and muscle pattern reconstruction by synergies**. **(A)** Examples of cursor trajectories executed by subject 2 in EMG-control (EC, *green)* and their reconstruction using four synergies (SR, *red*). Each column shows a trial to a different target (*gray circle*). Markers indicate the time of target acquisition. **(B)** Corresponding cursor displacements in x- and y- force directions for each trial. **(C)** Rectified and filtered EMG traces recorded during each trial (*gray area*) and their reconstruction (*red*) by the four subject-specific synergies shown in Figure 2B. Vertical dashed lines indicate the time of target acquisition. **(D)** Time varying synergies coefficients (color coded as in Figure 2B) for each trial. **(E)** Polar plot of the directional tuning of the four synergies shown in Figure 2B.

**Figure 4:**
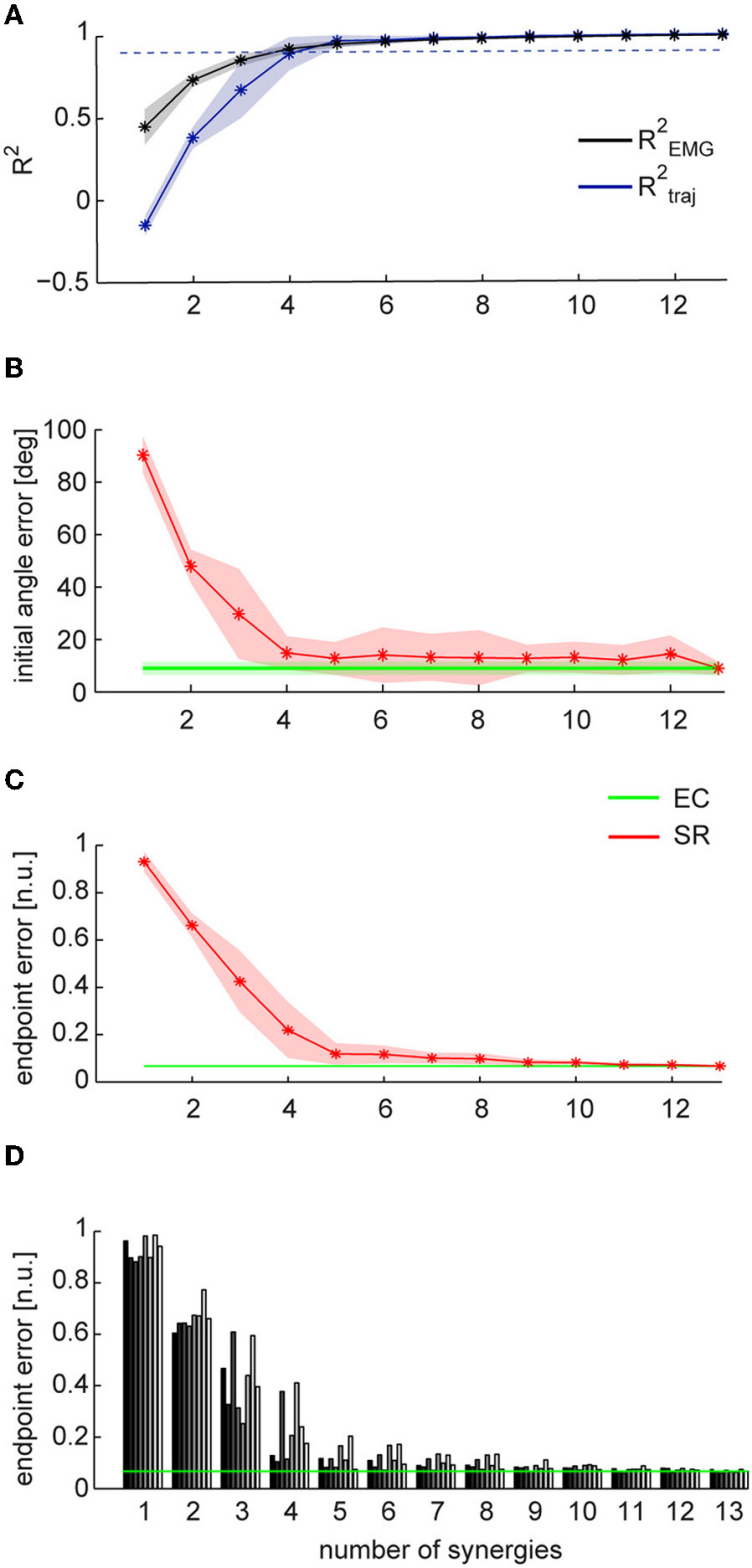
**Comparison between trajectories executed in EMG-control and trajectories reconstructed using synergies. (A)** Mean fraction of EMG data variation explained by synergies (R^2^_EMG_, *black*) and mean fraction of EC trajectory variation explained by SR trajectories (R^2^_traj_, *blue*) as a function of the number of synergies (*n* = 8, shaded areas indicate SD, dashed line indicate 0.9 R^2^). **(B)** Mean initial angle error for EC and SR trajectories as a function of the numbers of synergies. **(C)** Mean endpoint error (normalized to target distance from the origin, *n.u.: normalized units*) for EC and SR trajectories as a function of the number of synergies.**(D)** Endpoint error of SR trajectories for individual subjects (bars with different gray levels) as a function of the number of synergies. Average endpoint of EC trajectories across subject is indicated by the *green* line.

To address the question whether a small set of muscle synergies not only explains a large fraction of the variation of the muscle activity but can also generate the forces necessary to perform an isometric reaching task accurately, we compared the trajectories performed by human subjects during EMG-control (EC) with the trajectories reconstructed using the subject-specific synergies (synergy-reconstructed, SR). Figures 3A,B illustrate examples of EC (*green*) and SR (*red*) trajectories for 8 different targets in one subject. Figure 3C shows the corresponding filtered EMGs traces (*gray area*) and their synergy reconstruction (*red line*) using four synergies (Figure 2B). These synergies adequately captured the muscle patterns across directions and muscles. For each direction a different combination of synergy coefficients is used (Figure 3D), e.g., in direction 0° (1st column) synergy 2 and 4 are recruited, whereas in direction 225° (6th column) synergies 1 and 3 are recruited. The directional tuning of all four synergies is well captured by a cosine functions (Figure 3E, see also Borzelli et al., 2013; Gentner et al., 2013). The SR trajectories show a high similarity to the EC trajectories in each of the eight target directions. To quantify similarity, we first computed the fraction of EC trajectory variation (R^2^_traj_) explained by trajectories reconstructed using for each subject a number of synergies adequately capturing the EMG data variation (R^2^_EMG_, see Methods) which varied across subjects between 4 and 5 (see Table 1). On average across subjects (*n* = 8) we found that SR trajectories reconstructed accurately EC trajectories (mean R^2^_traj_ value 0.96 ± 0.03 *SD*, range: 0.89–0.98, Table 1). We then assessed the mean similarity of EC and SR trajectories as a function of the number of synergies (*N*) used for the reconstruction (Figure 4A, averages across subjects, *n* = 8). Five synergies were sufficient to reconstruct the EC trajectories with an average R^2^_traj_ value larger than 0.9 (mean R^2^_traj_ values were 0.89 and 0.97, for *n* = 4 and 5, respectively).

**Table 1:** **Number of synergies estimated from EMG-patterns in EMG-control (Experiment 1), fraction of EMG data variation explained, and performance measures for individual subjects of EMG-control trajectories (EC) and trajectories reconstructed using synergies (SR)**.

**Subject Id**	**1**	**2**	**3**	**4**	**5**	**6**	**7**	**8**	**Mean ± *SD***
Number of synergies	5	4	5	4	5	5	5	4	4.6 ±0.5
R^2^_EMG_	0.93	0.95	0.95	0.95	0.93	0.96	0.93	0.93	0.94 ±0.01
R^2^_traj_ (EC reconstruction by SR)	0.97	0.98	0.98	0.97	0.96	0.98	0.89	0.94	0.96 ±0.03
Initial angle error [deg] (SR)	10.5	9.8	17.0	13.4	30.1	6.0	30.5	8.0	15.7 ±9.6
Initial angle error [deg] (EC)	14.4	8.1	9.3	7.7	8.1	6.1	9.7	7.4	8.9 ±2.5
Endpoint error (SR)	0.12	0.10	0.11	0.11	0.17	0.11	0.20	0.17	0.14 ±0.04
Endpoint error (EC)	0.08	0.06	0.06	0.07	0.07	0.06	0.07	0.07	0.07 ±0.005

To assess the performance of the synergy reconstruction at the beginning of the movement, we then compared the initial angle error of EC and SR trajectories (Table 1 and Figure 4B). Using for each subject a number of synergies adequately capturing the EMG data variation (see Table 1), we did not find any significant differences between the mean initial angle error of EC and SR trajectories (Table 1, *p* = 0.087, *t*-test, *n* = 8). Figure 4B (*red*) shows the mean initial angular error of SR trajectories as a function of the number of synergies (*N*), averaged across subjects (*n* = 8), in comparison with the mean error of EC trajectories (*green*). While the trajectories reconstructed with 4 synergies had a significantly larger angle error than EC trajectories (*p* = 0.027, *n* = 4, *n* = 8, *t*-test with mean values ± *SD* of 15.1 ± 5.5°, with respect to 8.9 ± 2.5° in EC), there was no significant difference between the error of EC trajectories and the error of trajectories reconstructed using 5 synergies (*p* = 0.14, *n* = 5, *n* = 8, *t*-test, mean value ± *SD*: 14.7 ± 10.2).

To assess the performance of the synergy reconstruction at the end of the movement and to make a direct comparison to the results of de Rugy et al. (2013), we also estimated the endpoint error of the trajectories (Table 1 and Figure 4C). When comparing the endpoint error of the EC and SR trajectories we found similar results to those of de Rugy et al. (2013). Using for each subject a number of synergies adequately capturing the EMG data variation (see Table 1) we found a significant difference between the mean endpoint errors of EC and SR trajectories (*p* = 0.00015, Wilcoxon ranksum test, *n* = 8). Comparing the endpoint errors of EC and SR trajectories as a function of the number of synergies, we also found a significant difference between average endpoint errors for both four and five synergies (*p* = 0.0002 and *p* = 0.0003, for *n* = 4 and *n* = 5, Wilcoxon ranksum test, *n* = 8. Mean ± *SD* 0.22 ± 0.12 and 0.12 ± 0.05 for *n* = 4 and *n* = 5, respectively, compared to 0.067 ± 0.005 during EC). However, the analysis of individual subjects revealed a high inter-subject variability (Figure 4D). There was no significant difference between the endpoint error of EC and SR trajectories using 5 synergies for three out of eight subjects (*p* = 0.3481, *p* = 0. 2883, and *p* = 0. 9589 for subjects 2, 4, and 8, with *n* = 5) and for one subject using 4 synergies (*p* = 0.0764 for subject 1).

In summary, we found on average a high similarity between cursor trajectories under EMG-control and trajectories reconstructed using a set of synergies which adequately explained EMG data variation. Moreover, the mean initial angle error, indicative of the accuracy of the feed-forward commands, was not different between trajectories executed in EMG-control and trajectories reconstructed using synergies. However the mean endpoint error, more sensitive to feedback control, was larger for trajectories reconstructed using synergies than for trajectories executed using EMG-control of individual muscles.

### Synergies reconstruction of cursor trajectories during force-control

**Figure 5:**
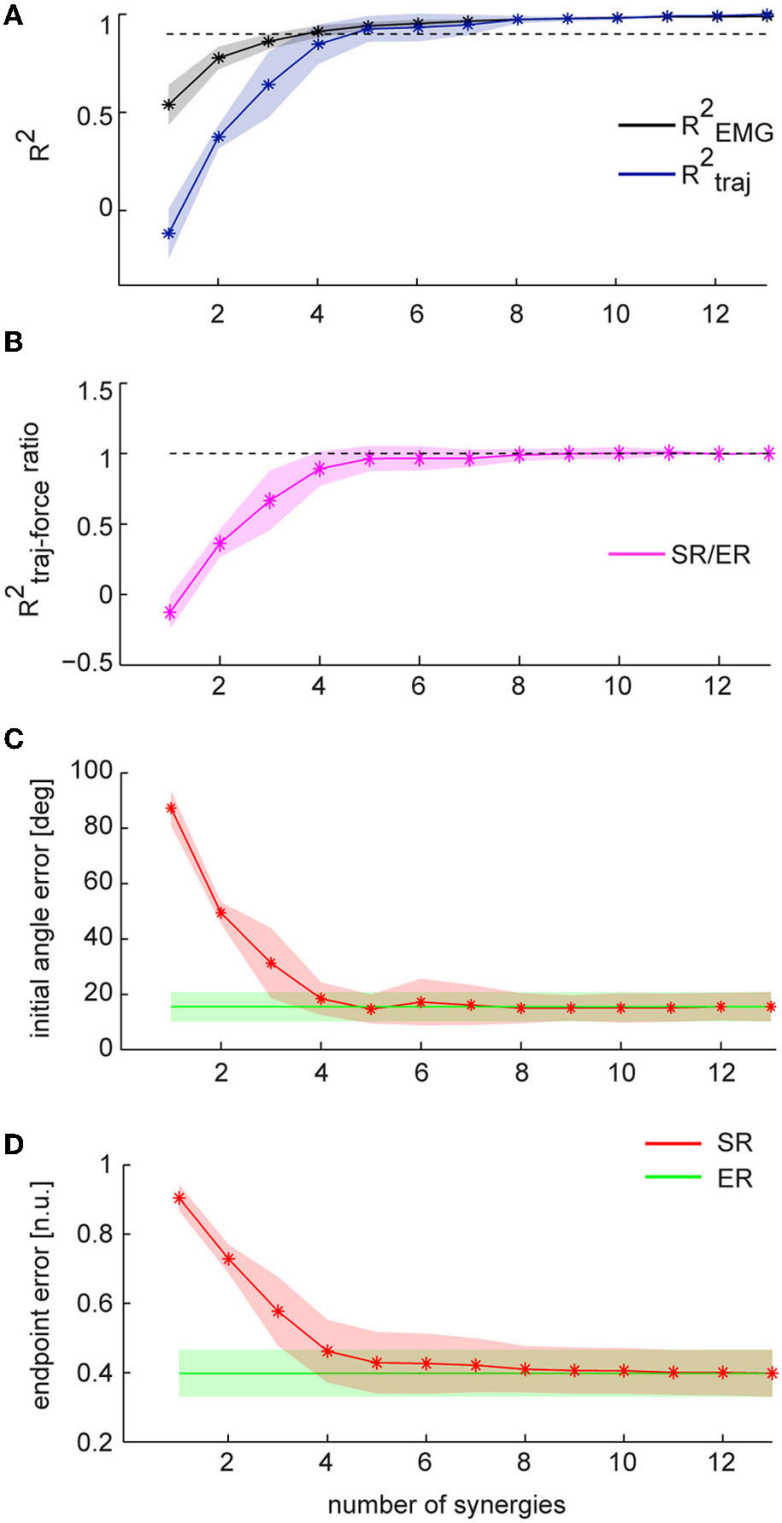
**Comparison between trajectories reconstructed using individual muscles and synergies during task performance in force-control**. Trajectories reconstructed using EMG data (ER) are shown in *green*; trajectories reconstructed using synergies (SR) are shown in *red*. **(A)** Mean fraction of EMG data variation explained by synergies (R^2^_EMG_, *black*) and mean fraction of ER trajectory variation explained by SR trajectories (R^2^_traj_, *blue*) as a function of the number of synergies (*n* = 8, shaded areas indicate SD, dashed line indicate 0.9 R^2^). **(B)** Mean of the ratio between the fraction of FC trajectory variation (R^2^_traj-force_) explained by SR trajectories and the fraction of FC trajectory variation explained by ER trajectories. Dashed line indicate a ratio of 1. **(C)** Mean initial angle error for ER and SR trajectories as a function of the numbers of synergies. **(D)** Mean endpoint error (normalized to target distance from the origin) for ER and SR trajectories as a function of the number of synergies.

As inaccuracies in the EMG-to-force mapping could be corrected by online adjustments in EC while inaccuracies in the synergy-to-force mapping could not be corrected in the offline reconstruction by synergies, we also compared the trajectories reconstructed using the EMG data (ER) recorded while human subjects performed the isometric reaching task in force-control (FC) with the trajectories reconstructed using subject specific synergies (SR). We quantified the similarity of ER trajectories with SR trajectories by computing the fraction of ER trajectory variation explained by SR trajectories (R^2^_traj_, Table 2 and Figure 5A). When we used, for each subject, a number of synergies adequately explaining EMG data variation (Table 2), we found, across subjects (*n* = 8), a mean R^2^_traj_ value of 0.88 ± 0.08 *SD* (Table 2). When we considered the mean R^2^_traj_ value as a function of the number of synergies (Figure 5A), we found that the mean R^2^_traj_ value was 0.85 ± 0.10 with four synergies and 0.93 ± 0.06 with five. We also compared how ER and SR trajectories reconstructed FC trajectories by computing the ratio of the fraction of FC trajectory variation explained (R^2^_traj−force_) by SR and by ER trajectories (Table 2 and Figure 5B). Selecting a number of synergies, for each subject, adequately capturing EMG data variation we found a mean R^2^_traj−force_ ratio (SR/ER) of 0.92 ± 0.11 (*SD*). Averaging the R^2^_traj−force_ ratio across subjects as a function of the number of synergies (Figure 5B) we found that 5 synergies reached a value of 0.96 ± 0.09 (*SD*). Thus, during force-control, we found a high similarity between the trajectories reconstructed using the entire set of recorded muscles and those reconstructed using only a small number of synergies.

**Table 2:** **Number of synergies estimated from EMG-patterns in force-control (Experiment 1), fraction of EMG data variation explained, and performance measures for individual subjects of trajectories reconstructed using EMGs (ER) and synergies (SR) during force-control**.

**Subject Id**	**1**	**2**	**3**	**4**	**5**	**6**	**7**	**8**	**Mean ± *SD***
Number of synergies	4	4	4	4	4	5	6	4	4.4 ± 0.7
R^2^_EMG_	0.91	0.96	0.92	0.93	0.91	0.96	0.94	0.91	0.93 ± 0.02
R^2^_traj_ (ER reconstruction by SR)	0.94	0.95	0.75	0.91	0.89	0.96	0.76	0.90	0.88 ± 0.08
Initial angle error [deg] (SR)	23.1	8.9	21.3	20.6	16.8	10.1	34.0	13.3	18.5 ± 8.2
Initial angle error [deg] (ER)	22.2	9.0	14.3	12.2	23.4	10.7	18.5	14.0	15.5 ± 5.3
Endpoint error (SR)	0.47	0.34	0.57	0.39	0.56	0.40	0.57	0.41	0.46 ± 0.09
Endpoint error (ER)	0.44	0.33	0.41	0.33	0.51	0.36	0.43	0.36	0.40 ± 0.06

We then compared the initial angle error of ER and SR trajectories. Figure 5C shows the average errors for ER trajectories and SR trajectories using, for each subject, a number of synergies which captured EMG data variation adequately. We found no significant differences between mean errors for ER and SR trajectories (*p* = 0.26, *t*-test, *n* = 8, mean ± *SD* 18.5 ± 8.2° and 15.5 ± 5.3 for ER). We also found no significant difference between the mean angle error of ER trajectories and the mean error of SR trajectories reconstructed using 4 synergies (*p* = 0.17 for *n* = 4 synergies, *t-test*, *n* = 8, with mean values of 18.4 ± 5.9° for SR and as above for ER). Finally, comparing the endpoint error of ER to SR trajectories (Table 2 and Figure 5D) we found no significant differences between the average errors using the subject-specific number of synergies (*p* = 0.19, *n* = 8, Wilcoxon ranksum test, mean ± *SD*: 0.46 ± 0.09 for SR and 0.40 ± 0.06 for ER) as well as when using 4 synergies for all subjects (*p* = 0.10, *n* = 8, Wilcoxon ranksum test, mean ± *SD* for SR: 0.46 ± 0.09). Thus individual muscles and synergies showed similar performance during force-control.

### Performance during synergy-control, EMG-control, and force-control

**Figure 6:**
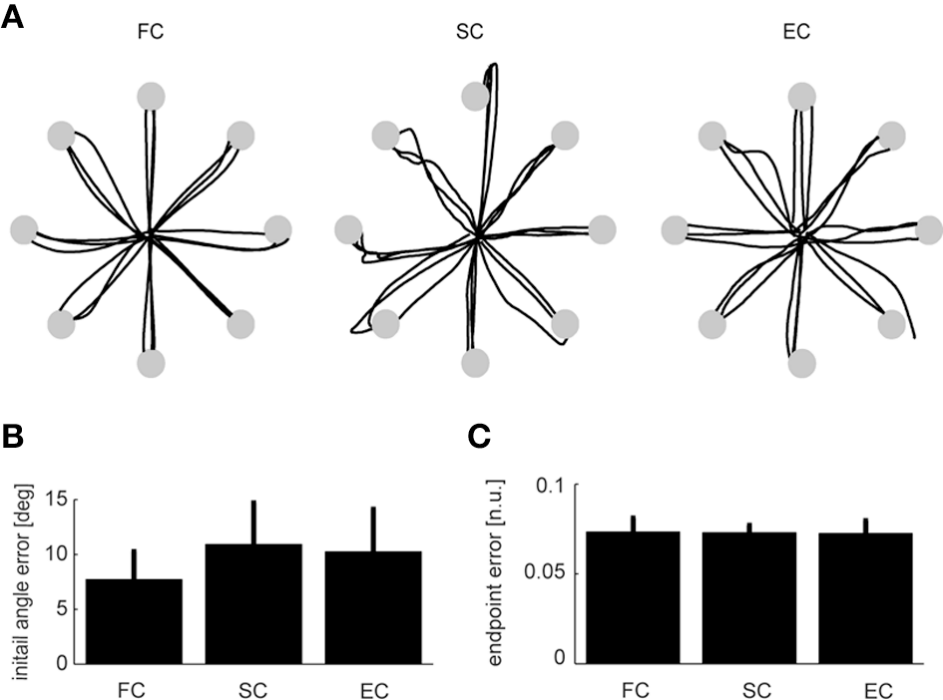
**Comparison between trajectories during force-control, synergy-control, and EMG-control. (A)** Examples of cursor trajectories during the execution of individual trials to the eight different targets during force-control (FC, *left*), synergy-control (SC, *middle*), and EMG-control (EC, *right*) for subject 9. Cursor trajectories are shown up to the time of arrival at the target (gray disks) or until the end of the trials in the target was not reached. **(B,C)** Averages across subjects (Experiment 2, *n* = 6) of the mean initial angle error **(B)** and mean endpoint error **(C)** over all the trials in the first block performed in FC, SC, and EC. Error bars indicate SD across subjects. All pairwise comparisons were not significant (see text).

We then investigated how well subjects were able to control the cursor directly with the synergy activation estimated online from the recorded EMGs, i.e., in synergy-control (SC) mode, by comparing their performances in FC, SC, and EC. In SC we used for each subject a number of synergies adequately capturing EMG data variation (Table 3). Subjects were able to control the cursor in SC and EC mode immediately after FC. Figure 6A shows examples of the trajectories for the first three movements in each of the eight directions performed in each control mode by one subjects. In these examples, all trials except one (bottom right target in EC) were successful. On average across subjects only 2.8 ± 3.4% (*SD*) of the trials in SC and 3.5 ± 6.7% of the trials in EC were unsuccessful while all trials were successful in FC for all subjects. The differences in the fraction of unsuccessful trials between all conditions were not significant (*t*-test, *n* = 6; SC–EC: *p* = 0.72; FC–SC: *p* = 0.10; FC–EC: *p* = 0.26).

**Table 3:** **Number of synergies extracted from EMG-patterns in force-control (Experiment 2), fraction of EMG data variation explained, and performance measures for individual subjects of trajectories executed in force-control (FC), synergy-control (SC), and EMG-control (EC)**.

**Subject Id**	**9**	**10**	**11**	**12**	**13**	**14**	**Mean ± *SD***
Number of synergies	5	5	7	6	5	5	5.5 ± 0.8
R^2^_EMG_	0.92	0.93	0.97	0.93	0.95	0.92	0.93 ± 0.02
Initial angle error [deg] (FC)	6.4	12.0	6.3	7.9	8.9	4.8	7.7 ± 2.5
Initial angle error [deg] (SC)	8.7	13.9	18.4	9.4	10.7	8.8	11.6 ± 3.8
Initial angle error [deg] (EC)	6.6	14.9	12.6	7.0	12.2	5.0	9.7 ± 3.9
Endpoint error (FC)	0.070	0.075	0.078	0.068	0.074	0.065	0.072 ± 0.005
Endpoint error (SC)	0.075	0.075	0.073	0.060	0.070	0.067	0.070 ± 0.006
Endpoint error (EC)	0.064	0.061	0.077	0.069	0.065	0.070	0.067 ± 0.006

We first assessed the subjects' performance by comparing initial angle errors (Figure 6B). There was no significant difference in angle error for the pairwise comparisons of the three different control modes (FC–SC: *p* = 0.067; SC–EC: *p* = 0.15; FC–EC: *p* = 0.13, *t*-test, *n* = 6, mean values ± SD for FC, SC, and EC, respectively, were: 7.7 ± 2.5, 11.6 ± 3.8, and 9.7 ± 3.9°). We then investigated the endpoint error (Figure 6C). We also found no significant difference in the endpoint error for the pairwise comparisons of the three different control modes (FC–SC: *p* = 0.94; FC–EC: *p* = 0.89; SC–EC: *p* = 0. 91, *t*-test, *n* = 6, mean values ± *SD* for FC, SC, and EC, respectively, were: 0.072 ± 0.005, 0.070 ± 0.006, and 0.067 ± 0.006). In summary, during all three control modes subjects were able to control the cursor accurately and there were no significant differences in initial angular error and endpoint error.

## Discussion

As in many previous studies, we found that a small number of muscle synergies explained a large fraction of the variation of the muscle patterns recorded during different task conditions. In this study, however, we focused on the question whether a small number of muscle synergies can accurately generate the forces involved in the task and, ultimately, whether muscle synergies can be used to perform the task effectively. We first tested whether trajectories of a virtual mass displaced by EMG activity recorded from 13 arm muscles during an isometric reaching task could be reconstructed with similar accuracy using the combinations of the same muscle activities into a small number of muscle synergies identified by NMF. Our results showed that the trajectories reconstructed using 4–5 synergies were as accurate as the trajectories obtained by displacing a virtual cursor according to the hand force estimated from EMGs recorded from the entire set of muscles when considering the initial movement direction error but not in terms of endpoint error. However, these results were not consistent across subjects, as for some subjects we found no difference in endpoint error using 4 or 5 synergies. We then assessed whether the availability of feedback in EMG-control mode could explain the lower endpoint accuracy of the synergies by comparing the reconstructions of the trajectories executed in force-control using synergies and using the entire set of muscles. Indeed, in force-control cursor movements depended only on the applied force and did not provide any information on the inaccuracy likely present in the EMG-to-force and synergy-to-force mappings used for the reconstruction. Trajectories reconstructed using synergies were not significantly different from trajectories reconstructed using individual muscles suggesting that the difference between the trajectories generated during EMG-control and their reconstruction by synergies observed in some subjects were due to online adjustments performed during the EMG-control mode. Finally, we explicitly tested whether human subjects were able to control a virtual cursor and to perform successfully a force reaching task by synergy recruitment. In synergy-control mode the cursor movement depended only on the portion of the recorded EMGs that could be reconstructed by synergy combinations. Subjects were able to control the cursor in synergy-control mode immediately after switching from force-control mode and there were no significant differences in performance between the three control modes.

As mentioned in the Introduction, several studies have provided evidence supporting the hypothesis that movements are controlled by a limited set of modules or muscle synergies. However, most of these studies focused on how well synergies describe muscle patterns, showing that a small number of synergies capture a large fraction of the muscle pattern variation across task conditions and often that such synergies are robust across different tasks, but they did not directly address the question whether synergies can be used by the CNS to effectively accomplish a task. Thus, in order to validate the synergy hypothesis it must be demonstrated that a small number of synergies are sufficient to generate the forces or movements necessary for accurate task performance. Two recent studies have addressed the functional role of the muscle synergies underlying postural responses to stance perturbations in cats (Torres-Oviedo et al., 2006) and in humans (Chvatal et al., 2011) by using NMF to simultaneously extract synergies from EMG data and kinetic data (contact forces, center of mass accelerations). Such functional muscle synergies could explain both muscle activation patterns and kinetic data in a range of postural configurations and in different types of responses, suggesting that muscle synergies are responsible for the control of specific biomechanical functions shared across task conditions. Consistent functional roles of muscle synergies have also been demonstrated in dynamic simulations of human pedaling and walking (Raasch and Zajac, 1999; Neptune et al., 2009; Allen and Neptune, 2012). Identification of functional muscles synergies, however, relies on the assumption of a linear relationship between EMGs and kinetic variables which might be valid only in limited conditions. Similarly, accurate forward dynamic simulations using muscle synergies depend on many musculoskeletal parameters that are difficult to validate and require fine-tuning of the muscle excitation patterns. Thus, these studies do not provide direct evidence that a small set of muscle synergies is sufficient for achieving accurate task performance.

A recent study by de Rugy et al. (2013) investigated the relation between synergies and task performance in humans by comparing force trajectories generated under EMG-control during an isometric reaching task, similar to the one used in the present study but involving only five wrist muscles, with the trajectories reconstructed using muscle synergies. One advantage of this experimental approach is that task performance depends on a well-defined linear transformation of the recorded EMGs, even if such linear mapping is not an accurate estimate of the real EMG-to-force mapping. de Rugy and collaborators found that four synergies on average explained more than 90% of the EMG data variation but they reconstructed cursor trajectories with a much higher endpoint error than the error of the trajectories executed in EMG-control. The authors claimed that synergy decomposition introduces substantial task space errors and concluded that applying synergy decomposition onto a set of available muscles appears of little use to best reconstruct the motor output in task space.

In the present study we have addressed the issue of whether muscle synergies can accurately reconstruct and generate forces in a number of ways. First, we performed the same analysis that de Rugy and collaborators performed on the wrist system (using EMG activity recorded from five muscles) on the more complex arm system (using EMG activity recorded from 13 muscles). We compared the endpoint error of cursor trajectories during EMG-control of a reaching task with the endpoint error of trajectories reconstructed using synergies. We found, on average across subjects, that a small number of synergies were not sufficient to reach the same endpoint accuracy as during EMG-control, thus replicating for the arm the results by de Rugy et al. (2013) for the wrist. However, investigation of individual subjects showed that this result was inconsistent across subjects. For half of the subjects synergy reconstruction using 4 or 5 synergies was sufficient to reach the same performance as during EMG-control. Moreover, as our subjects were instructed to reach the target with a fast reaching movement and were not required to minimize endpoint error, we also compared the entire cursor trajectories and the initial directional error and we found no significant differences between EMG-control and reconstruction with a number of synergies adequately explaining the EMG data.

Second, as the comparison of EMG-control and synergy reconstruction may be biased by the use of online feedback, we also compared EMG and synergy reconstruction of cursor trajectories generated by recorded forces. In EMG-control cursor trajectories are generated by forces estimated using EMG signals and not by the recorded forces and subjects were able to exploit feedback to correct inaccuracies in the estimated EMG-to-force mapping. However, inaccuracies in the synergy-to-force mapping may require different corrections unavailable in the offline synergy reconstruction. We therefore argue that in order to conclude that synergy decomposition decreases task performance with respect to individual muscle control, one needs either to compare experimental conditions in which online corrections to both EMG-to-force and synergy-to-force inaccuracies are possible (as in our second experimental protocol), or to compare the reconstructions, using individual muscles and synergies, of trajectories executed in force-control mode, in which neither EMG-to-force nor synergy-to-force inaccuracies can be corrected. When we compared the trajectories reconstructed using synergies and using individual EMGs using data collected while the task was performed in force-control mode we did not find any significant difference. These results indicate that the trajectories reconstructed using synergies are not as accurate as the trajectories executed in EMG-control because of online feedback corrections during EMG-control.

Third, we directly demonstrated the effectiveness of a small number of synergies in generating the forces involved in a reaching task by showing that subjects were able to control the cursor accurately with the synergies. We compared performances when subjects controlled the cursor with synergies to performances in EMG-control and force-control. Remarkably, subjects were able to perform the task immediately after switching from force-control to synergy-control and they did not show significant differences in initial angle error and endpoint error between the three control models. These results show that subjects were able to control a cursor in a reaching task using synergies with similar performance as during force-control and EMG-control.

Finally, de Rugy et al. (2013) examined the wrist system, which has a relatively low muscle redundancy, and it might not require synergistic control. They also drew similar conclusions analyzing arm muscle pattern that were simulated as to generate the target forces, using an EMG-to-force mapping derived from a biomechanical model, with minimal summed squared muscle activations (Fagg et al., 2002). They justified the use of simulated data for assessing the task efficacy of muscle synergies also in the more complex arm system with the observation that wrist simulated data show a fraction of variance explained by synergy decomposition and endpoint error of trajectories reconstructed using synergies similar to those obtained with experimental data. However, the similarity between simulated and experimental data in the weakly redundant wrist system might be due to the lack of synergistic control in such system. In contrast, simulated data might have a different synergy decomposition than experimental data collected in the arm system if this is controlled synergistically. In fact, a recent study by Borzelli et al. (2013) investigating muscle patterns of the arm underlying isometric force generation has shown that the estimated minimum effort recruitment of individual muscles does not adequately capture the observed muscle activation patterns. Thus, using simulated muscle patterns de Rugy et al. (2013) did not test whether a small number of synergies could achieve good task performance in the arm system. Not surprisingly the simulated data had a high dimensionality and the synergy decomposition resulted in higher aiming errors than individual muscles. However, if the data had been generated by combinations of a small number of synergies their decomposition into the same number of synergies would have achieved the same task performance as individual muscles. Thus, the results of these simulations, because they depend on the assumptions made for data generation, cannot lead to any conclusion on whether the CNS does employ synergies for simplifying control.

The number of synergies used for reconstructing cursor trajectories executed in force- and EMG-control and for projecting recorded EMG data in synergy-control is a critical parameter that has a major effect on the task space accuracy. We selected the number of synergies, a free parameter in the NMF decomposition of the EMG data, comparing the EMG data variation accounted by different number of synergies (R^2^_EMG_). We considered two criteria used in many previous studies of muscle synergies: the minimum number of synergies with R^2^_EMG_ over 90% (Tresch et al., 1999; Ting and Macpherson, 2005; Torres-Oviedo et al., 2006; Roh et al., 2012; Delis et al., 2013) and the number of synergies at which the slope of the R^2^_EMG_ curve has a change in slope (d'Avella et al., 2003; Cheung et al., 2005; d'Avella et al., 2006; Tresch et al., 2006; Delis et al., 2013). When the number of synergies selected by the two criteria did not match we chose the largest number to ensure the best reconstruction of the EMG data when analyzing task performance. However, both criteria depend on *ad-hoc* thresholds and they do not ensure the selection of the correct number of synergies. Importantly, we noticed that adding a single synergy may sometimes have a small effect on R^2^_EMG_ but a large effect on the quality of the reconstruction of EMG-control trajectories using synergies (R^2^_traj_) (see Figure 4D, subject 3). Thus, the lower task performance of the trajectories reconstructed using synergies, in addition to the lack of feedback driven adjustments to synergy-to-force inaccuracy discussed above, might also be due to the inappropriate selection of the number of synergies for some of the subjects. However, the number of synergies selected with our criterion did allow to perform the task in synergy-control with accuracy similar to that of force- and EMG-control, suggesting that the criterion was adequate and that the lower performance of synergy-reconstructed trajectories executed in EMG-control was mainly due to the lack of appropriate feedback. Finally, task performance in synergy-control could be used as a new criterion for the selection of a number of synergies, especially for synergy-based myoelectric control applications. It would simply require testing an increasing number of synergies and selecting the number that ensures performance comparable to that obtained with individual muscles.

In conclusion, the investigation of a 2-dimensional force reaching task demonstrated that a complex arm muscle system can be effectively controlled by a small number of synergies. Our results suggest that muscle synergies are employed by the CNS to cope with the high number of degrees-of-freedoms in the musculoskeletal system and to simplify movement coordination. However, the fact that we did not find evidence for any significant reduction in performance using muscle synergies cannot definitively prove whether or not synergies are actually employed by the CNS. Further insights into the synergy hypothesis may be gained by testing subjects' adaptation to perturbation. In a recent study (Berger et al., 2013) we have shown that adaption to virtual surgeries, i.e., perturbation of the muscle-to-force mapping, depends on the compatibility of the surgery with the synergies. Human subjects adapted strikingly faster after compatible virtual surgeries, in which a full range of movements in the task space could be achieved recombining the initially identified synergies, than after incompatible virtual surgeries, for which new or modified synergies would be required. Muscle synergies might thus allow for faster adaptation to perturbation and to environmental demands. Comparing adaptation to perturbation directly under synergy-control and EMG-control might therefore shed further light into possible control strategies employed by the CNS. Synergy-control could moreover be useful for achieving intuitive simultaneous and proportional control of myoelectric prostheses (Jiang et al., 2009, 2013), and robot arms (Artemiadis and Kyriakopoulos, 2010) and for the development of novel diagnostic tools and rehabilitation approaches (Safavynia et al., 2011). Specifically, rehabilitation exercises in a virtual environment with synergy-control might promote recovery of movement skills in stroke patients by facilitating the recruitment of spared muscle synergies (Cheung et al., 2009, 2012) and the re-organization of altered ones (Roh et al., 2013).

### Conflict of interest statement

The authors declare that the research was conducted in the absence of any commercial or financial relationships that could be construed as a potential conflict of interest.
